# Synthesis, Antimicrobial, Moisture Absorption and Retention Activities of Kojic Acid-Grafted Konjac Glucomannan Oligosaccharides

**DOI:** 10.3390/polym11121979

**Published:** 2019-12-01

**Authors:** Lin Song, Wancui Xie, Yukun Zhao, Xinyao Lv, Huanbin Yang, Qingpei Zeng, Zuoxing Zheng, Xihong Yang

**Affiliations:** 1 College of Marine Science and Biological Engineering, Qingdao University of Science & Technology, Qingdao 266042, China; 2 Key Laboratory for Biochemical Engineering of Shandong Province, Qingdao 266042, China; 3 Wuqiong food Co., Ltd., Raoping 515726, China

**Keywords:** konjac glucomannan oligosaccharides, kojic acid, synthesis, antibacterial, moisture retention

## Abstract

Kojic acid (KA) with antibacterial activities produced by fermentation was grafted onto konjac glucomannan oligosaccharide (KGO) composed of glucose and mannose linked by β-1,4 glycosidic bonds. A novel KGO derivative, konjac glucomannan oligosaccharide kojic acid (KGOK) possessing both moisture retention and antibacterial activities was synthesized. The structure of KGOK was characterized and analyzed by thermogravimetric analysis (TG), XRD, UV–vis absorption, FTIR, and ^1^H NMR. The analysis results suggest that KA was linked to the KGO molecular chain through a covalent bond, and the reaction site of KA was the methylol group. The studies demonstrate that KGOK maintained the excellent moisture absorption and retention properties of KGO and the good antibacterial activities of KA. The minimum inhibitory concentration (MIC) of KGOK is 2 mg/mL for *Staphylococcus aureus*, *Staphylococcus epidermidis*, *Shewanella putrefaciens*, and *Salmonella enterica*, while its MIC is 3 mg/mL for *Escherichia coli*. The multi-functionality of the KGOK synthesized from natural sources provides a theoretical foundation for their potential applications in the preservation of food, beverage, aquatic, and cosmetic products.

## 1. Introduction

Natural polysaccharides and oligosaccharides have the characteristics of non-toxicity, biodegradability, and significant water retention, which have been widely used in aquatic products [1,2,3,4,5,6,7]. Konjac glucomannan (KGM) is a high-molecular-weight natural plant polysaccharide extracted from the rhizome of *Amorphophallus konjac*. It is composed of glucose and mannose linked by β-1,4 glycosidic bonds at a molar ratio of 1:1.6 with 5–10% of the acetyl group [3,8]. KGM has been approved as GRAS (generally recognized as safe) for food use by the U.S. Food and Drug Administration (FDA) [9,10]. KGM has been widely used in aquatic products, food and beverages, medicine, and other fields [11,12]. Studies have shown that KGM can improve the gel properties of surimi and has a good protective effect on the myofibrillar protein during frozen storage [13,14].

For many food applications, KGM has some undesirable properties such as high viscosity, limited water solubility, and low permeability due to the presence of the acetyl groups and high molecular weight. Through deacetylation and depolymerization, KGM can be converted into konjac glucomannan oligosaccharide (KGO), which is endowed with almost 100% absorbability, in relation to KGM, and thus can entirely dissolve both in water and physiological solution [3,15]. Although natural KGM and KGO have many good properties, these functionalities can be further improved through certain molecular modifications. Various forms of KGM derivatives obtained by physical, chemical, and enzymatical modifications have been used in a range of different applications, including biodegradable films, medical and pharmaceutical materials, functional foods, and aerogel [10]. Thus, the modification of KGM and KGO gains much interest from numerous researchers because of their better performance in food applications.

Kojic acid (KA), 5-hydroxy-2-hydroxymethyl-1,4-pyrone, is a weakly acidic secondary metabolite produced by aerobic fermentation of *Aspergillus*, *Acetobacter*, and *Penicillium*. KA has a variety of biological activities, including antibacterial, antifungal, antiviral, and antioxidant activities [16,17,18,19]. Therefore, KA and its derivatives have attracted much attention for their variety of biological activities.

In aquatic product preservation, the most common challenges are water retention and bacterial inhibition. Foodborne pathogens and spoilage bacteria such as *Staphylococcus aureus*, *Shewanella putrefaciens*, *Escherichia coli*, and *Salmonella enterica*, which are common strains found in aquatic products [20,21,22]. *Staphylococcus epidermidis* is a bacterium that breeds on the epidermis of organisms. Most of them are non-pathogenic bacteria and a few can cause diseases. *S. aureus* is a commensal and opportunistic pathogen that can cause contamination of food products during preparation and processing [23]. *S. putrefaciens* is a marine, gram-negative bacterium which plays a role in the food industry as a spoilage bacterium of marine fish and chicken due to its ability to produce volatile sulfides, amines, and the fishy-smelling compound trimethylamine [24]. As one of the oligosaccharides, KGO has excellent moisture retention properties. Combining KA’s antimicrobial activity with KGO’s moisture retention properties in one compound would be an ideal approach to solving the common issues in aquatic product preservation, such as food spoilage and freeze dried consumption [25]. Fish and shellfish have high water content, rich nutrients, and strong autolytic enzyme activity, which make them easy to corrupt and deteriorate, shortening the shelf life of aquatic products [26]. Common food preservatives in the market include chemical preservatives (potassium sorbate, sodium diacetate, etc.) and biological preservatives (propolis, chitosan, plant polysaccharide, etc.). Chemical preservatives have some side effects and long-term use is harmful to human health. However, the biological preservation method is safer and could meet people’s consistently increasing requirements for food. As a new type of preservation product, KGOK possessed not only antimicrobial activity but also moisture retention properties. KGOK was superior to other preservatives, both biological and chemical ones [25,26].

This study was to explore the possibility of synthesizing a konjac glucomannan oligosaccharide-kojic acid derivative (KGOK) from KA and KGO and using it as a potential solution to the problems in aquatic product preservation. Both KGO and KA are natural food additives used in food products for improving food quality and safety. Although konjac oligosaccharides have excellent moisture retention properties, they are not bacteriostatic. KGOK having both excellent moisture retention and bacteriostatic properties was synthesized by grafting a kojic acid group onto KGO. KGOK was characterized by thermogravimetric analysis (TG), XRD, UV–visible spectroscopy, FTIR, and ^1^H NMR spectroscopy. The analysis results suggest that KA was linked to the KGO molecular chain through a covalent bond, and the reaction site of KA was the methylol group.

## 2. Materials and Methods

### 2.1. Materials

KGO and KGM were purchased from Xi’an Quanao Biotechnology Co. Ltd. (Xi’an, Shaanxi, China). KA was obtained from Shanghai Yuanye Biotechnology Co. Ltd. (Shanghai, China). Chitosan (CTS) and chitosan oligosaccharide (COS) were purchased from Qingdao Maidier Bioengineering Co., Ltd. (Qingdao, Shandong, China). Hyaluronic acid (HA) was purchased from Qufu Shengjiade Biotechnology Co., Ltd. (Qufu, Shandong, China). The remaining reagents are all commercially available in analytical grades and were used without further purification. The microorganisms were provided by Qingdao Entry–Exit Inspection and Quarantine Bureau, China. Strain number: *Staphylococcus aureus* (ATCC 25923), *Salmonella enterica* (CGMCC 1.16091), *Shewanella putrefaciens* (CICC 22940), *Escherichia coli* (ATCC 51755), and *Salmonella enterica* (ATCC 25241).

### 2.2. Synthesis of Konjac Glucomannan Oligosaccharides/Kojic Acid

**Figure 1:**
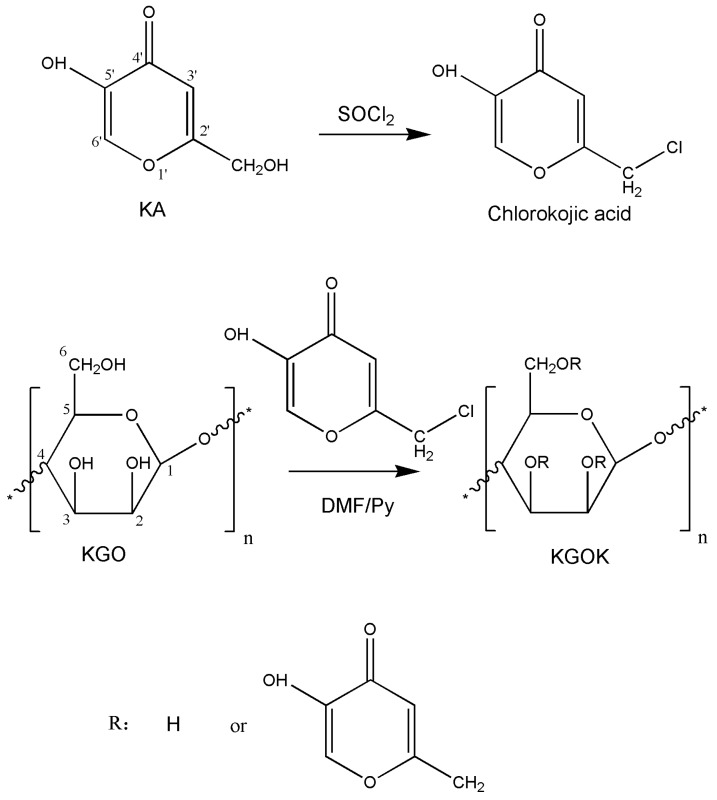
Reaction scheme for the synthesis of konjac glucomannan oligosaccharides/kojic acid.

The first step is to convert kojic acid into chlorokojic acid by mixing kojic acid with thionyl chloride in water at 0 °C to form a precipitate, and the precipitate was washed with petroleum ether until the filtrate was colorless to obtain chlorokojic acid [23]. In the second step, KGO and chlorokojic acid were separately dissolved in round-bottom flasks containing dimethylformamide (DMF) at a molar ratio of 1:2 and stirred at room temperature until completely dissolved. The KGO solution was slowly added dropwise to the chlorokojic acid solution, and an appropriate amount of pyridine (Py) was added dropwise thereto, and the reaction was stirred magnetically for 24 h. Acetone was added to the solution to obtain a precipitate, which was then separated from the solution by vacuum filtration. The precipitate was the initial product of KGOK [24]. The initial product was subjected to Soxhlet extraction with acetone for 24 h, and the extracted solid was dissolved in deionized water, dialyzed against deionized water for 24 h using a 1 kDa dialysis bag. It was then concentrated by rotary evaporation and finally was lyophilized to obtain a purified KGOK. The product was synthesized by two-step reactions, and the reaction conditions and purification methods were simple and effective (Figure 1).

### 2.3. Characterization

The basic structure and substitution position of the samples were determined by five characterization methods. Thermogravimetric analysis (TG) for the samples was performed on a NETZSCH TG 209F1 Libra thermogravimeter (NETZSCH Group, Germany). The samples were put into desiccators containing saturated potassium carbonate solution for two days of equilibration. TG was carried out at the heating rate of 10 °C/min under an N_2_ atmosphere in the temperature range of 30–600 °C. Crystallinity measurements (XRD) were made using a Rigaku D/max Full-automatic X-ray diffractometer (Rigaku Corporation, Japan) with Cu Kα radiation at a voltage of 40 kV and a current of 40 mA. The scanning rate was 5°/min from 5° to 80° at room temperature. UV–visible absorption spectroscopy (Aoyi Corporation, China) determination of KA, KGO, and KGOK was carried out in the wavelength range of 190–600 nm and with a beam width of 2 nm. The Fourier-transform infrared (FTIR) spectrum was recorded on a Nicolet iS10 instrument (Thermo Scientific, MA, USA). Samples were prepared as KBr pellets at wavenumber range 4000–400 cm^−1^; ^1^H NMR spectra were obtained on a Bruker AC-500 instrument (Bruker Corporation, MA, USA). Samples were dissolved in heavy water (D_2_O). The inhibition rate (antibacterial activity) was determined on a Multiskan FC instrument (Thermo Scientific, MA, USA) at the wavelength of 620 nm.

### 2.4. Antibacterial Activities

The antibacterial activities of KA and KGOK were determined by a broth microdilution method [25]. The testing protocols are described below.

About 50 μL of glycerol stock of each target bacterial strain was inoculated into tryptic soy broth (TSB) in a shake flask and the shake flask was cultured under optimum culture conditions [27]. The culture solution was centrifuged to obtain a precipitate of the bacterial cells. The precipitate was then suspended in sterile 0.9% physiological saline to obtain the bacterial stock suspension.

The samples were made into a stock suspension at a concentration of 16 mg/mL using TSB. The samples were filtered and sterilized with 0.22 μm membrane. In addition, the sterilized TSB medium was cooled to room temperature before the sample was added.

The antibacterial activity was determined by the difference in absorbance by adding different concentrations of the samples to the 96-well microtiter plate. The initial sample solution was added to the first well of the 96-well microtiter plate, and the other concentrations of the sample were diluted by gradient dilutions. The bacterial stock was added and mixed well into each well. The uninoculated microtiter plate only containing the sample solutions was used as a blank control. The final sample concentrations in the microtiter plate were 8, 6, 4, 3, 2, 1, 0.5, and 0 mg/mL. The initial absorbance of the solution in a 96-well microtiter plate at a wavelength of 600 nm was measured using a microplate reader, and the plate was placed in a bacterial incubator under optimum culture condition. *E. coli, S. aureus, S. enterica, S. epidermidis, and S. putrefaciens* were cultured at 37 °C and 150 r/min for 24 h. The absorbance was measured again at the same wavelength. The difference between the two optical density (OD) values was recorded as OD_δ_, and the difference in absorbance of the control group was recorded as OD_δ0_, and the difference in absorbance of the other solutions to be tested was recorded as OD_δx_. The minimum inhibitory concentration (MIC) was indicated as the lowest concentration at which the inhibition rate was not less than 99%. Each sample was measured three times, and the inhibition rate (antibacterial activity) of the samples was calculated by the following formula:

Inhibition rate = (OD_δ0_ − OD_δx_)/OD_δ0_ × 100%. (1)

### 2.5. Moisture Absorption and Moisture Retention Properties

Moisture absorption and moisture retention rate measurement refers to the scheme of Yang et al. [26]. The samples were ground into a powder and dried in an oven at 100 °C for 4 h. The samples (0.5 g) were put into desiccators that contained a saturated ammonium sulfate solution (81% relative humidity, RH) and a saturated potassium carbonate solution (43% RH), respectively, and the desiccators were kept at room temperature for 84 h. The moisture absorption ability (R_a_) was evaluated by the percentage increase of weight in samples after equilibration in the desiccators:

R_a_ = (W_n_ − W_0_)/W_0_ × 100%, (2)

where W_0_ and W_n_ were the weights of the samples before and after they were put into the desiccators, respectively. Samples were consecutively tested at different time points.

The samples (0.5 g each) were ground into powders in a mortar and pestle and dried in an oven at 100 °C for 4 h. Water (0.05 g) was added to each sample. The samples were then put into desiccators that contained water saturated with silica gel, ammonium sulfate, and potassium carbonate, respectively, and the desiccators were kept at room temperature for 72 h. The moisture retention ability (R_h_) was evaluated by the percentage of residual water in the wet sample:

R_h_ = (H_n_ − H_0_) × 100%, (3)

where H_0_ and H_n_ were the weights of water in the samples before and after they were put into the desiccators, respectively. Samples were consecutively tested at different time points.

## 3. Results

### 3.1. Characterization of KGOK

**Figure 2:**
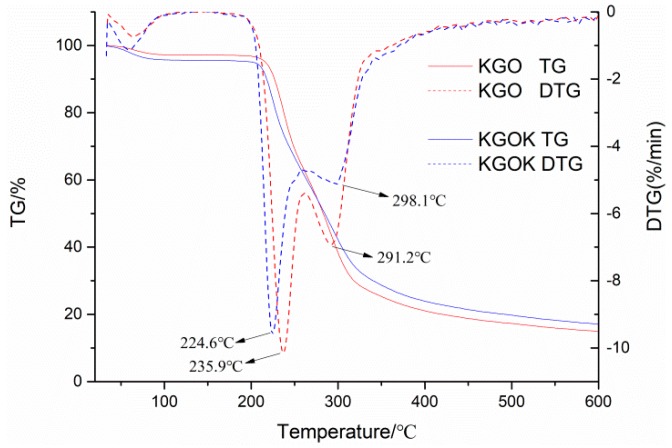
Thermogravimetric curves of konjac glucomannan oligosaccharide (KGO) and konjac glucomannan oligosaccharide kojic acid derivative (KGOK).

Thermogravimetric analysis (TG) and derivative thermogravimetric analysis (DTG) have been widely used to evaluate the thermal properties of materials and to show the mechanism of material weight reduction due to controlled heating. Figure 2 shows the TG and DTG curves of KGO and KGOK. Both KGO and KGOK involved three stages of weight loss as temperature increased. The first stage was related to the dehydration in the temperature range of 30–100 °C. The water weight losses of KGO and KGOK were 2.82% and 4.40%, respectively. It was reported that KGOK possesses better moisture retention than KGO at 43% RH [28]. The second stage was attributed to the disintegration of molecule chains. The peak disintegration temperatures of KGO and KGOK were 235.9 and 224.6 °C, respectively. The third stage was related to the degradation of saccharide rings [29,30]. In the third stage, the peak temperature in DTG increased from 291.2 °C (KGO) to 298.1 °C (KGOK) with the degradation rate decreased due to the covalent bond of KGOK between the molecule chains and the KA substituent. Thus, KGOK was thermally more stable than KGO.

**Figure 3:**
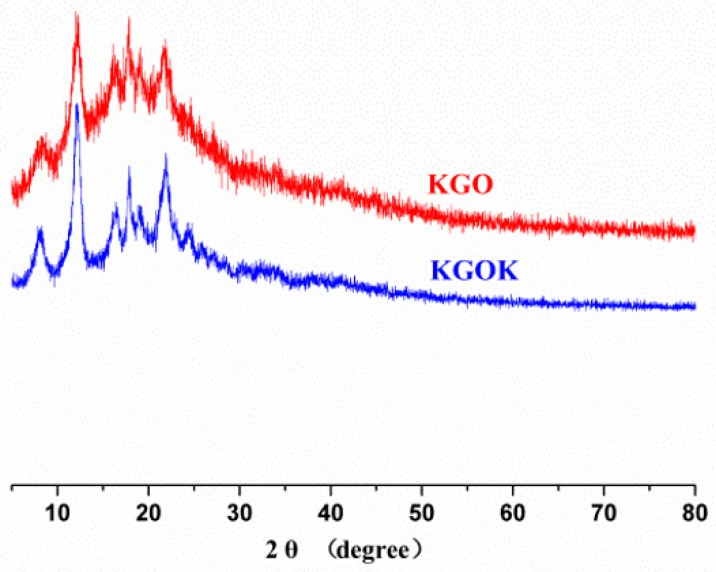
XRD patterns of KGO and KGOK.

The thermal stability of KGOK was judged by XRD based on whether it was due to crystal form, because a slight change in crystalline structure could cause significant alteration in thermal stability [27]. The XRD patterns of KGO and KGOK are shown in Figure 3. The KGO and KGOK exhibited some peaks at around 2θ = 8.2°, 12.4°, 16.2°, 17.8°, 19.1°, and 24.6°. This result shows that no new crystal shape was formed by addition of kojic acid. Meanwhile, the peak of KGO was wider and weaker, indicating that kojic acid hindered the signal of the original crystal. TG result shows that the crystal of KGOK is more stable than that of KGO. This result of XRD was consistent with that of TG.

**Figure 4:**
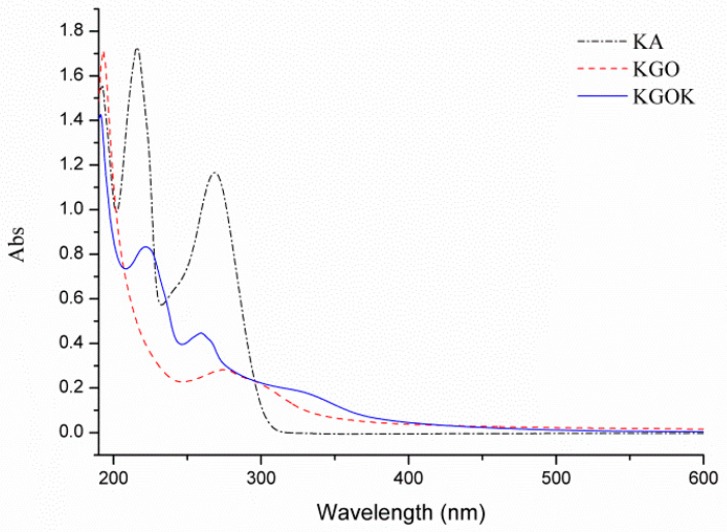
UV−vis spectroscopy of kojic acid (KA), KGO, and KGOK.

In the UV–vis spectra (Figure 4) of KA, KGO, and KGOK, broad absorption bands between 200 and 300 nm were observed for all three samples. According to some reports in the literature, the broad absorption band of KGO might be ascribed to the C=O group [31]. In the UV spectrum of KA, the obvious absorption peaks appeared at 216.2 and 268.2 nm. The two absorption bands of KGOK at 221.6 and 259.4 nm indicate the presence of a 5-hydroxypyranone group. These bands were assigned to intraligand n–π* and π–π* transitions of the chromophoric C=O group [18], indicating that KA had been grafted onto the KGO molecule.

**Figure 5:**
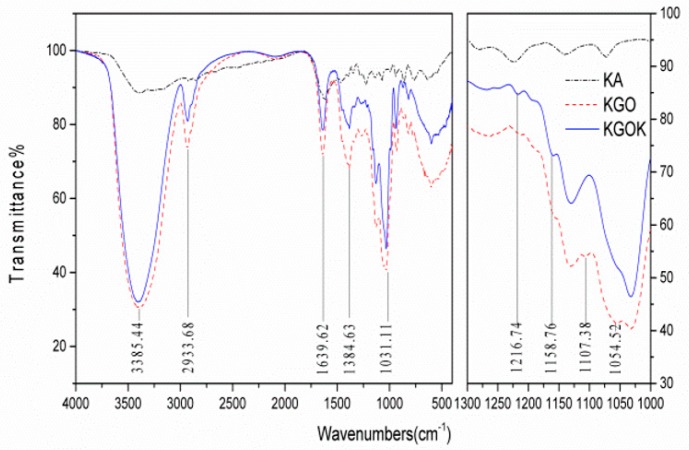
FTIR spectrum of KA, KGO, and KGOK in the wavelength region 4000–400 cm^−1^.

FTIR spectroscopy of KA, KGO, and KGOK is shown in Figure 5. IR spectrum of KGO showed absorption bands around 3385.44, 2933.68, 1384.63, and 1031.11 cm^−1^ corresponding to the stretching of the –OH, –CH_2_, –CH, and C–O–C bridge, respectively. The peak at 1639.32 cm^−1^ was assigned to the stretching of the intramolecular hydrogen bond [32]. The absorption peaks at 1054.52 and 1107.38 cm^−1^ were the C–O stretching vibrations in the primary hydroxyl groups (C_6_ of KGO) and the secondary hydroxyl groups (C_2_, C_3_ of KGO), respectively. In the IR spectrum of KA, the absorption peaks at 1657.62, 1579.81, and 1224.56 cm^−1^ were the characteristic absorption of the stretching vibration of C=O, C=C, and C–O–C in 5-hydroxypyranone, respectively [18]. In the IR spectrum of KGOK, the substitution of KA groups caused the breakage of the hydrogen bond of KGO, and as a result, the intensity of the IR spectrum absorption peak was weakened, and the peak shape was narrowed. The substitution of the KA group produced an inducing effect such that the absorption peak of the –OH group shifted to a high wave number to 3404.80 cm^−1^. Attenuation of the absorption peak at 1054.52 and 1107.38 cm^−1^ indicated that KA reacted with the hydroxyl group on the KGO molecular chain. The newly emerging absorption peak at 1158.76 cm^−1^ was the C–O stretching vibration of the tertiary hydroxyl group (C_5_ of KA) on the KA group, which proved that KA was linked to the KGO molecular chain through a covalent bond, and also indicated the reaction site of KA was methylol group (C_2′_ of KA). The band at 1216.74 cm^−1^ was broadened and shifted from the corresponding features of chlorokojic acid that could be due to covalent bonding of the kojic acid residues onto KGO [33].

**Figure 6:**
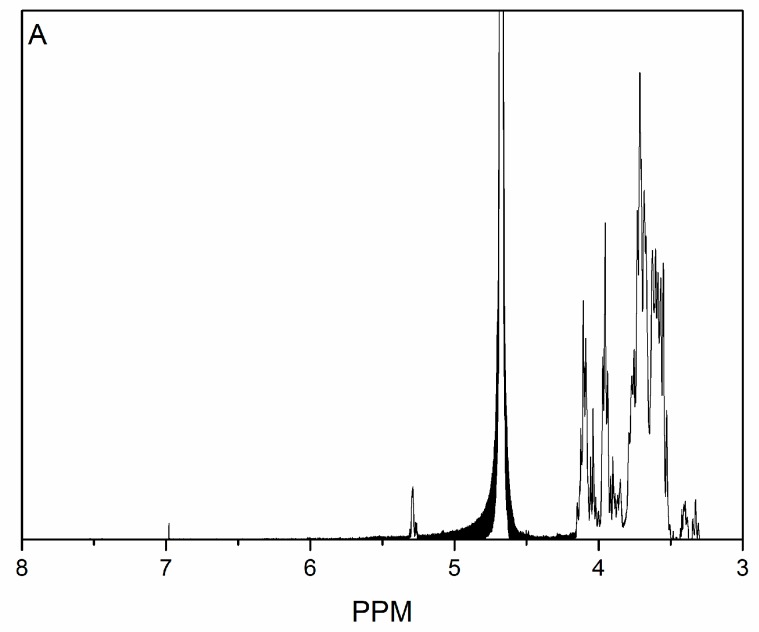

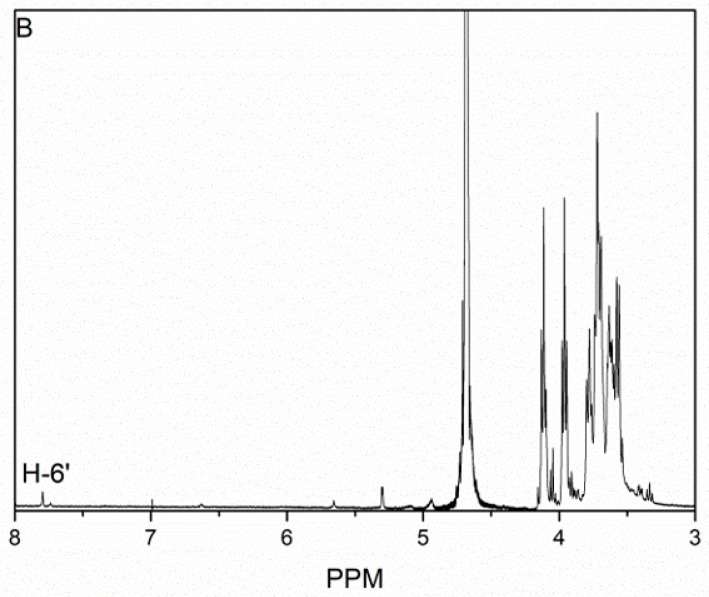
^1^H NMR spectrum of KGO (**A**) and KGOK (**B**).

The ^1^H NMR spectra of KGO and KGOK in D_2_O solvent are shown in Figure 6. The solvent proton resonates at 4.7. In Figure 6B, signals of the newly formed resonance signal appeared at 7.80 (H-6′) ppm. The new peaks were assigned to the protons of kojic acid residues bound to the polysaccharide [18].

### 3.2. Antimicrobial Activity

The minimum inhibitory concentration (MIC) of KA against *S. aureus*, *S. epidermidis*, *S. putrefaciens*, and *S. enterica* was 0.5 mg/mL, while the MIC against *E. coli* was 1 mg/mL (Table 1). KGOK had the MIC of 2 mg/mL for *S. aureus*, *S. epidermidis*, *S. putrefaciens*, and *S. enterica*, while it had the MIC of 3 mg/mL for *E. coli* (Table 1). KGOK was effective against many bacteria and thus could be used to prevent the growth of some pathogenic or spoilage bacteria in aquatic products.

### 3.3. Moisture Absorption and Retention Properties

**Figure 7:**
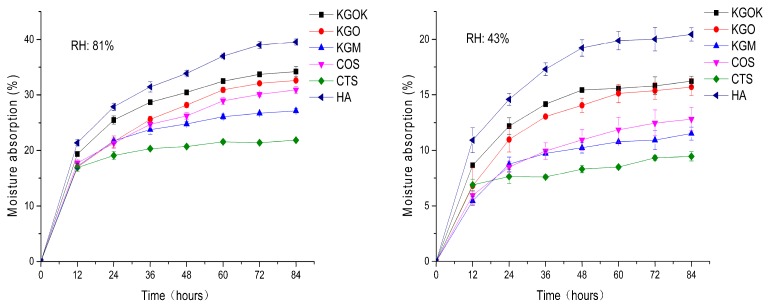
Moisture absorption abilities of KGOK, KGO, KGM, chitosan oligosaccharide (COS), chitosan (CTS), and hyaluronic acid (HA).

Figure 7 shows the change in moisture absorption of KGOK, KGO, KGM, COS, CTS, and HA within 84 h. At 81% and 43% RH, the moisture absorption of all samples increased, and the moisture absorption rate increased rapidly in the first 12 h and then the growth rate slowed down. HA had the highest moisture absorption, while KGOK had the best moisture absorption among all sugar samples. By comparing the moisture absorption rates of KGO, KGM, COS, and CTS, it can be found that the moisture absorption of the oligosaccharide was better than that of the polysaccharide. At 81% RH, the moisture absorption of KGOK reached 34.21%, while that of KGO was 32.62%. At 43% RH, the moisture absorption of KGOK reached 16.24%, while that of KGO was 15.69%. Thus, KGOK still has the excellent moisture absorption of KGO.

**Figure 8:**
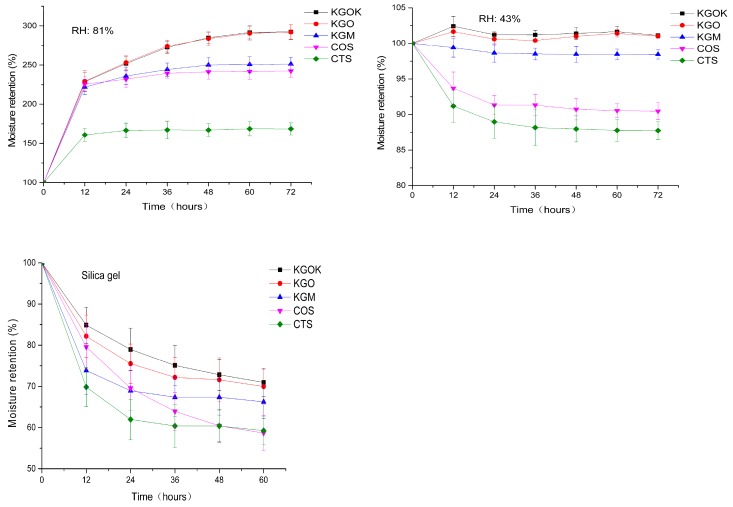
Moisture retention abilities of KGOK, KGO, KGM, COS, and CTS.

Figure 8 shows the change in moisture retention of KGOK, KGO, KGM, COS, and CTS at 81% and 43% RH, and with silica gel, respectively. At 81% relative humidity, the moisture retention of all samples showed an increasing trend, in which the moisture retention rate of KGOK and KGO was essentially the same, 292.46% and 292.05%, respectively, at 72 h, and both were superior to other polysaccharides. At 43% RH, the moisture retention rates of KGOK and KGO were basically maintained at the initial state, which was 101.13% and 101.02% at 72 h, respectively. The moisture retention rate of KGM showed a slow downward trend, which was 98.44% at 72 h. The moisture retention rate of COS and CTS decreased significantly, which was 90.47% and 87.75% at 72 h, respectively. In the silica gel experiment, the moisture retention rate of all samples showed a downward trend, in which KGOK had the highest water retention rate. The final moisture retention rate from high to low was 70.96% for KGOK, 69.96% for KGO, 66.25% for KGM, 58.67% for COS, and 59.25% for CTS. From the results, the moisture absorption ability of KGOK is higher than that of KGO, KGM, COS, and CTS, whereas it is lower than that of HA. We deduced that the moisture retention competence of the KGOK may be due to its chemical structure. From previous studies, the water retention mechanism of hyaluronic acid depends on its helical columnar structure and hydroxyl group [34]. Biological polysaccharides which possess polyhydrophilic groups can bond with water in the form of hydrogen bonds [35]. KGOK, as a monosaccharide derivative, has such a chemical structure, resulting in its superior functionality in water retention.

## 4. Discussion

The product was characterized by TG, XRD, UV–vis absorption, FTIR, and ^1^H NMR, which confirmed that KGO reacted with KA to form KGOK. TG showed that KGOK was thermally more stable than KGO. XRD showed that the crystal form of KGOK is more stable than that of KGO. This result of XRD was consistent with that of TG. The other analysis results suggest that KA was linked to the KGO molecular chain through a covalent bond, and the reaction site of KA was the methyl group. Hydrophilicity is a major feature of KGO [36]. Compared with KGO, the antimicrobial activity of KGOK was significantly enhanced. Consistent with our experimental results, previous studies have shown that KA has significant antimicrobial properties [37]. As a food additive, the amount of kojic acid must be controlled within the safe range. In this study, we found that the antimicrobial activity of KGOK is lower than that of KA. We concluded that it was related to the addition of a bacteriostatic agent. Previous studies also have shown that KA’s antibacterial activity against gram-negative bacteria is higher than that against gram-positive bacteria [37]. There were also some differences in the antimicrobial activity against gram-positive bacteria (*S. aureus, S. epidermidis*) and gram-negative bacteria (*S. putrefaciens, E. coli,* and *S. enterica*) for KGOK [38]. As shown in Table 1, KGOK’s minimal inhibitory concentration on *E. coli* (gram negative) was higher than its MIC for *Staphylococcus* (gram positive). The results above indicated that KGOK’s antibacterial activity against gram-negative bacteria is higher than that against gram-positive bacteria. On one hand, the peptidoglycan layer of gram-positive bacteria is composed of networks with plenty of pores, which allow KGOK molecules to come into the cell without difficulty. On the other hand, the 5-hydroxypyranone group was introduced along the molecular chain, which can form complexes with various metal ions, which is important in cell membranes [18]. The moisture absorption and retention activities of KGOK and KGO were basically the same, and the oligosaccharide with low molecular weight has better moisture retention capacity than the polysaccharide with high molecular weight [39]. Furthermore, the solubility of oligosaccharides is higher than that of polysaccharides. However, in our study, the solubility of KGOK and KGO were basically the same. The result was consistent with TG that the water weight loss of KGOK was more than that of KGO at the temperature range of 30–100 °C. KGOK still possesses the excellent moisture retention capacity of KGO.

In summary, KGO is a kind of oligosaccharide with excellent water retention properties, and kojic acid is a natural compound that is widely present in fermented products and has good antibacterial properties. The kojic acid-grafted konjac glucomannan oligosaccharide, KGOK, has good antibacterial activity against bacteria including *S. aureus*, *S. epidermidis*, *S. putrefaciens*, *E. coli*, and *S. enterica*. KGOK also has excellent water absorption and retention properties. Given such desirable multi-functionalities, KGOK should have broad application potential in the food, beverage, and cosmetic applications.

## Figures and Tables

**Table 1:** The minimal inhibitory concentration (MIC) of KA and KGOK.

Minimal Inhibitory Concentration (MIC) (mg/mL)		
Strain Name	KA	KGOK
*Staphylococcus aureus*	0.5	2.0
*Staphylococcus epidermidis*	0.5	2.0
*Shewanella. putrefaciens*	0.5	2.0
*Escherichia coli*	1.0	3.0
*Salmonella enterica*	0.5	2.0
